# Educational differences in years lived with disability due to mental and substance use disorders: a cohort study using nationwide Norwegian and Danish registries

**DOI:** 10.1186/s12889-024-20064-0

**Published:** 2024-09-20

**Authors:** Nanna Oerslev Weye, Oleguer Plana-Ripoll, Carl Michael Baravelli, Emilie E. Agardh, Lode van der Velde, Jonas Minet Kinge, Ann Kristin Skrindo Knudsen

**Affiliations:** 1 Department of Clinical Epidemiology, Aarhus University and Aarhus University Hospital, Aarhus, Denmark; 2 Department of Disease Burden, Norwegian Institute of Public Health, Bergen, Norway; 3 National Centre for Register-based Research, Aarhus University, Aarhus, Denmark; 4 Department of Global Public Health, Karolinska Institutet, Stockholm, Sweden; 5 Department of Health Management and Health Economics, University of Oslo, Oslo, Norway; 6 Centre for Disease Burden, Norwegian Institute of Public Health, Bergen, Norway

**Keywords:** Disease burden, Educational gradient, Mental disorders, Substance use disorders

## Abstract

**Background:**

Findings from the Global Burden of Disease (GBD) study have shown that the burden of mental and substance use disorders is considerable, and unevenly distributed across demographic groups in the population. However, there is a lack of knowledge on how this burden differs by socioeconomic position. The aim of this study was to examine educational differences in years lived with disability (YLDs) from mental and substance use disorders among males and females in two high-income countries, taking comorbidity with other diseases into account.

**Methods:**

The study included all registered residents in Denmark and Norway from 2011 to 2021. Diagnostic information was retrieved from records in the Norwegian National Patient Registry (NPR) and the Danish Psychiatric Central Research Register (PCRR) and used as proxy measures for disorder prevalence. Demographical and educational information was taken from administrative registries. The YLD is a measure of the non-fatal health loss in the population and was calculated by multiplying the duration of a disorder with a disability weight (DW), scaled between 0 and 1. Information on remission and DWs were retrieved from the GBD study and other sources, and disorder specific DWs were averaged by severity levels and adjusted for comorbidity.

**Results:**

Educational gradients in YLD rates were found for mental and substance disorders overall, and for most of the specific disorders. The educational gradient was more pronounced for schizophrenia, intellectual disability and substance use disorders than for eating, anxiety, and affective disorders. Both higher YLD rates, and a larger attributed proportion of the total YLDs, were found for schizophrenia, intellectual disability, and substance use disorders in the groups with low versus high education. YLD rates for eating, anxiety, and affective disorders were more equal across educational levels, but constituted a smaller proportion of the total YLDs among the groups with low versus the groups with high educational level.

**Conclusion:**

Most of the disease burden related to mental and substance use disorders falls on those with the fewest years of education. This should be taken into consideration when public health targets aimed at improving mental health and reducing social inequalities in health are developed and implemented.

**Supplementary Information:**

The online version contains supplementary material available at 10.1186/s12889-024-20064-0.

## Key message

• The disease burden of mental and substance use disorder is unequally distributed by educational levels in both Denmark and Norway.

• The educational gradient in disease burden from mental and substance use disorders is more pronounced for schizophrenia, intellectual disability and substance use disorders, than for eating, anxiety, and affective disorders.

• Use of disease burden methodology on individually linked registry data extends the knowledge on the distribution of important causes of non-fatal health loss in different groups of the population.

**Supplementary Information:**

The online version contains supplementary material available at 10.1186/s12889-024-20064-0.

## Introduction

Knowledge about the distribution of poor health in the population is crucial for monitoring progress towards public health goals, and to prioritise and target research focus, expenditures, and interventions towards the groups with the highest need. The burden of disease concept, here defined as the total health loss in a population due to morbidity and premature mortality, was developed by the Global Burden of Disease project (GBD) and operationalised as the disability-adjusted life-year (DALY). Measures of disease burden have been important for comparing the impact of mortality and morbidity due to different diseases in different populations [1]. However, while GBD provides important knowledge about the distribution of disease burden across population groups aggregated by demographic characteristics (such as geographical location, age and sex), little is known about variation in disease burden across individual measures, such as socioeconomic position (SEP). The latter are important characteristics that can identify groups with particularly high risk for poor health outcomes.

The Nordic countries are among the worlds` most egalitarian countries. Core features of their strong welfare organisation include universal access to both higher education and health services, the redistribution of income through progressive taxes, and the existence of an economic safety net for those who are not able to fully participate in the labour market due to health problems. However, socioeconomic differences in health are present also in the Nordic countries, with some analyses even indicating that the socioeconomic gap in health is increasing [2, 3]. Hence, the equalizing of social differences in health is an important public health target also in the Nordic countries [4].

Numerous studies have demonstrated a strong inverse association between SEP and prevalence of mental and substance use disorders. This association is likely to be bidirectional, and a potential causal direction may vary between disorders [5, 6]. Persons with low SEP have been found to have a higher risk of developing a mental disorder [7–11], and mental disorders in childhood and early adulthood are found to be associated with lower SEP later in life [12, 13]. Educational level is a commonly used measure of SEP and is correlated with other measures like employment, income, and social status [14]. While mental and substance use disorders may affect the ability to initiate and complete higher education [15–18], low educational level may, through different mechanisms, also increase the risk of developing mental disorders [19, 20]. Three decades of analyses from the influential GBD study have shown that the burden of mental and substance use disorders in the Nordic countries is considerable [1, 21, 22], and in partiuclar among adolescents, young adults and the working-age population. However, there is a lack of knowledge on how the burden from mental and substance use disorders are distributed across groups in the population characterised by their SEP.

Denmark and Norway are two strong welfare states in the Nordic region, with comparable, high quality, complete and detailed registry-based information about educational level and health of their populations. The ability to link this information, gives a unique opportunity to examine socioeconomic differences in cause specific disease burden, and thus add to the knowledge provided by the GBD project. We have previously expanded on the GBD methods, by adjusting for observed comorbid conditions to estimate the non-fatal health loss from mental and substance use disorders using Danish individual-level registry data [23, 24]. In the present study, we will leverage on these methods and expand the scope to include Norwegian data, and information about educational level. Thus, the aim is to examine educational differences in disease burden from mental and substance use disorders among males and females in two high-income countries, taking comorbidity into account.

## Methods

### Overview over study design and calculation of disease burden

This registry-based cohort study included all registered residents in Denmark and Norway in the period January 1st, 2011, to December 31st, 2021. We examined educational differences in disease burden from mental and substance use disorders by linking individual data on educational level from administrative registries and diagnostic records from the secondary health care registries. In the GBD project, disease burden is operationalised as the disability-adjusted life-year (DALY). DALY is a summary measure of the healthy lifetime lost due to nonfatal health loss (years lived with disability (YLD)) and premature mortality (years of life lost (YLL)). However, since mental disorders rarely is recorded as the underlying cause of death, we have in this study focused on estimating educational differences in nonfatal health loss as measured by YLDs [25]. The YLD is calculated by multiplying the prevalence of a disorder with a disability weight, which measures the health-related disability of a given disorder on a scale from 0 to 1. Prevalence is calculated at any given point in time, taking duration and remission of the disease into account. Finally, and in contrast to the GBD project, which assumes independent comorbidity between diseases, we have adjusted for dependent comorbidity between mental disorders and other diseases in the calculation of non-fatal health loss.

### Study population

We included all individuals registered as residents in Denmark or Norway at any point between January 1st, 2011 to December 31st, 2021, equal to 7,199,758 individuals in Denmark and 6,152,364 individuals in Norway. Each individual was followed from birth, immigration, or January 1st, 2011, (whichever happened last) until death, emigration, or December 31st, 2021, (whichever happened first). Individuals were identified by their unique civil registration number, from the Danish Civil Registration System, established in 1968 [26] and the Norwegian National Population Register, established in 1964 [27]. These administrative registries contain information about date of birth (month of birth in Norway), sex, and place of residence. More information about the cohorts is given in supplementary methods (Supplementary material p.2).

### Educational level

Information on highest attained educational level was obtained for each year from the national education registers in Denmark [28] and Norway [29]. For individuals aged < 25 years, the highest educational level between the parents and the child was used, since they may not yet have reached their highest educational level. The educational systems are similar in Denmark and Norway, and the standard for classification have the same overall structure. Educational level was categorised according to the International Standard Classification of Education (ISCED) into low (compulsory education, ISCED 0–2), medium (upper secondary education or post-secondary non-tertiary education, ISCED 3–5), and high (tertiary education, ISCED 6–8), educational level. Educational level was considered a time-varying factor, allowing individuals to change educational level during the study period.

### Assessment of mental and substance use disorders

Information about diagnoses of mental and substance use disorders in the study period were retrieved from the Norwegian National Patient Registry (NPR) [30] and the Danish Psychiatric Central Research Register (PCRR) [31] and used as proxy measures for disorder prevalence. We used diagnostic records with information about primary and secondary diagnoses from health care visit in both inpatient, outpatient (including patients in specialist settings outside hospitals for Norway), or emergency room settings (Denmark only). Hence, if a person was recorded with a mental or substance use disorder either as a primary or secondary diagnosis in any of these settings, this person was considered a prevalent case of that disorder. NPR covers all public specialist health care, including outpatient, day patient, inpatient care, specialised interdisciplinary addiction treatment and mental health facilities for children, youths, and adults in Norway. The register contains individual-level information about primary and secondary diagnoses, which both were included in the current study, month and year of contact for the entire study population. All dates were set to 15th of the registered month. The PCRR contains individual-level information about primary and secondary diagnoses and date of contact for the entire study period in inpatient, outpatient, or emergency room settings. Visits to private practicing specialists in psychiatry and psychology are recorded without diagnostic information in the Danish National Health Service Register [32]. Consequently, in this study, we focus on mental disorders and substance use disorders diagnosed in secondary health care in Denmark and Norway.

Although individual follow-up started on January 1st, 2011, in both countries, prevalent mental disorders were identified from January 1st, 2008, to December 31st, 2010. Table 1 lists the included mental and substance use disorders. Diagnoses in the registries were coded according to the International Classification of Diseases, 10th version (ICD-10), and were grouped according to the categories and disorders used in the GBD project to employ the same disability weights, severity distribution and duration as in GBD (more details follow below). The disorders were grouped into three categories: (1) mental disorders with primarily onset in childhood (eating disorders, intellectual disability, autism spectrum disorders [ASD], attention-deficit hyperactivity disorder [ADHD] and conduct disorders), (2) mental disorders with primarily onset in adulthood (schizophrenia, bipolar disorder, major depressive disorder [MDD], anxiety disorders and personality disorder), and (3) substance use disorders (alcohol use [AUD], opioid use, cannabis use, cocaine use, amphetamine use and other drug use disorders). Detailed information about each disorder case definition, the GBD grouping of disorders by ICD-10 codes and minimum possible age of onset, is available in Supplementary Table 1.

### Assessment of comorbid general medical conditions

A range of general medical conditions (GMCs) have been found to contribute substantially to the disease burden from mental and substance use disorders [23]. Hence, to avoid inflated YLD rates due to this comorbidity, we adjusted for 33 GMCs in the analyses. Information about the GMCs were retrieved from the Danish and Norwegian Patient Registers [30, 32]. An individual was considered exposed to a specific GMC from the date of first diagnosis in the period January 1st, 2008, to December 31st, 2021, except for epilepsy, in which the date of second diagnosis was used as date of exposure. ICD-10 codes and disability weights for each GMC are available in Supplementary Tables 2–3.

### Calculating non-fatal disease burden – years lived with disability (YLD)

Years Lived with Disability (YLDs) is a measure of the disease burden attributed to non-fatal health loss. YLDs are calculated by multiplying the duration of a disorder with a disability weight (DW), which measures the health-related disability of a given disorder on a scale from 0 to 1. Duration was modelled using the observed date of diagnosis in secondary healthcare with GBD estimated remission rates for each mental and substance use disorder. For each date of contact for a disorder, an expected date of remission for that episode was calculated based on the date and the expected disorder duration following disease-specific remission rates from GBD. This approach results in two scenarios for persons with recurrent episodes of the same disorder: (1) either the episodes from two or more distinct contacts did not overlap – these were considered as distinct episodes of disease – (2) or the episodes overlapped in which case the exposure to the disorder was defined from the date of first diagnosis to the latest date of expected remission or end of follow-up (whichever came first). ASD, intellectual disabilities and all GMCs were assumed to be chronic conditions with no remission.

We employed the same DWs as in the GBD study [33, 34]. DWs were developed by the GBD study as an attempt to quantify the health-related disability associated with different health states, and hence to make different diseases comparable with each other, and with causes of premature death. They are based on responses from household and internet-based surveys conducted in nine countries from several parts of the world and range from 0 (being equivalent to full health) to 1 (being equivalent to death) [33, 34]. In a population, any mental or substance use disorder will be distributed across multiple severity levels (e.g. major depressive episode is classified into mild, moderate or severe level). Each of these severity levels are defined as individual health states and ascribed a unique disability weight (e.g. in the case of major depressive episode: 0.145 (mild), 0.396 (moderate), 0.658 (severe)). As we did not have information about the distribution across severity levels in our data, we generally used the same distribution across severity levels for mental and substance use disorders as in GBD [1, 35], except for asymptomatic levels. Due to our patient-based data, we assumed that asymptomatic levels would not be present in our study population. For the distribution of severity proportions of the GMCs, we also included sources beyond GBD to give estimates about the severity distribution, such as the Scottish Burden of Disease Study [36–38]. We used the average of the severity-specific disability weights, taking the proportion of cases of each severity into account [33, 35] (Supplementary Tables 1–3). Finally, and in contrast to the GBD project which assumes independent comorbidity between diseases, we accounted for the dependent comorbidity between mental and substance use disorders and other GMCs in the calculation of non-fatal health loss, employing the same methods as described in our previous publications [23, 24]. Disability weights were adjusted for comorbid mental and substance use disorders and GMCs using a multiplicative model so that no combination of disorders had a combined health state worse than death, represented by a disability weight equal or greater than one. The multiplicative model, therefore, accounts for individuals with multiple comorbid conditions to provide a more precise estimation of the overall health loss.

### Statistical analysis

Following Weye et al. [23, 24], we estimated YLDs for each disorder, stratifying on educational level and sex. YLDs were accumulated for the period 2011–2021 and estimated as the duration of a given disorder in years multiplied by the disorder-specific disability weight (summing up all individuals at the population level). Rates of YLDs were calculated as the absolute YLDs divided by person-years in the entire population. Although the distribution of age and sex is very similar in Denmark and Norway, results for the Norwegian population were standardised to the Danish population to obtain comparable age standardised rates (ASR). Confidence intervals (CI) were estimated using bootstrap with 500 iterations, which incorporated uncertainty in the disability weights and remission rates, by selecting a random number from a triangular distribution based on these two distributions in each iteration. The results are presented in coefficient plot figures, grouped for communicative reasons according to mental disorder with onset primarily in childhood or adulthood, or as substance use disorder, and as rates in supplementary tables. Further, the proportion of total YLDs attributed to specific disorders by educational level is presented in a bar graph. The results are presented separately for Denmark and Norway, but no formal comparison between the countries is done, due to the differences between the countries in terms of where in the health service specific disorders are being treated (see the Discussion for more details regarding this limitation of the data). All analyses were conducted in R version 4.1.2 and version 4.2.2. The figures were generated in Stata SE version 18.

## Results

Between January 1st, 2011, December 31st, 2021, data from the secondary health services show that there were 683,668 individuals diagnosed with mental or substance use disorders in a population of 6,152,364 individuals in Norway (11.2%) and 418,988 individuals diagnosed with mental disorders or substance use in a population of 7,199,758 individuals in Denmark (5.8%). In Norway, 40% of the population had attained higher education, versus 32% in Denmark (Table 1). The most common recorded diagnoses were major depressive disorder and anxiety disorders in both countries.

**Table 1:** Characteristics of study cohorts, cases of mental and substance use disorders^1^ and visits^2^, 2011–2021

	Denmark		Norway	
	N (%)		N (%)	
**Mean age at entry**	35.4		34.0	
**Sex**				
Males	3,613,027 (50)		3,108,653 (51)	
Females	3,586,731 (50)		3,043,711 (49)	
**Educational level**				
Low	1,303,491 (18)		1,096,370 (18)	
Medium	2,966,009 (41)		2,302,325 (37)	
High	2,269,227 (32)		2,441,086 (40)	
Missing	661,031 (9)		312,583 (5)	
**Disorder**	N (%)	Median visits (Q1-Q3)	N (%)	Median visits (Q1-Q3)
**Any mental or substance use disorder**	**418**,**988 (5.82)**	**4 (2–11)**	**683**,**668 (11.17)**	**7 (2–18)**
**Mental disorders with primarily onset in childhood**				
Eating disorders	23,045 (0.32)	3 (1–10)	27,842 (0.45)	6 (2–15)
Intellectual disability	22,211 (0.31)	2 (1–4)	25,864 (0.42)	4 (2–10)
Autism spectrum disorder	52,827 (0.73)	2 (1–6)	28,665 (0.47)	7 (2–16)
ADHD	78,814 (1.09)	3 (2–8)	102,781 (1.67)	7 (3–13)
Conduct disorder	4454 (0.06)	2 (1–3)	6656 (0.11)	3 (1–8)
**Mental disorders with primarily onset in adulthood**				
Schizophrenia	36,634 (0.51)	10 (3–30)	19,733 (0.32)	15 (3–43)
Bipolar disorder	29,088 (0.40)	5 (2–15)	45,374 (0.74)	8 (2–20)
Major depressive disorder	146,086 (2.03)	2 (1–5)	289,859 (4.71)	5 (2–11)
Anxiety disorder	107,188 (1.49)	2 (1–5)	255,464 (4.15)	4 (2–10)
Personality disorder	62,362 (0.87)	3 (1–8)	54,561 (0.89)	6 (2–16)
**Substance use disorders**				
Alcohol use disorder	49,283 (0.68)	2 (1–6)	100,097 (1.63)	2 (1–8)
Opioid use disorder	5194 (0.07)	3 (2–6)	22,230 (0.36)	13 (2–40)
Cannabis use disorder	26,378 (0.37)	2 (1–5)	32,518 (0.53)	4 (2–11)
Cocaine use disorder	4694 (0.07)	2 (1–4)	3656 (0.06)	2 (1–7)
Amphetamine use disorder	5411 (0.08)	2 (2–5)	20,044 (0.33)	3 (1–10)
Other drug use disorders	5924 (0.08)	2 (1–4)	26,223 (0.43)	2 (1–6)

^1^Mental disorders diagnosed in secondary health care in Denmark and Norway. ^2^ Median, 25th and 75th percentile number of visits

**Fig. 1:**
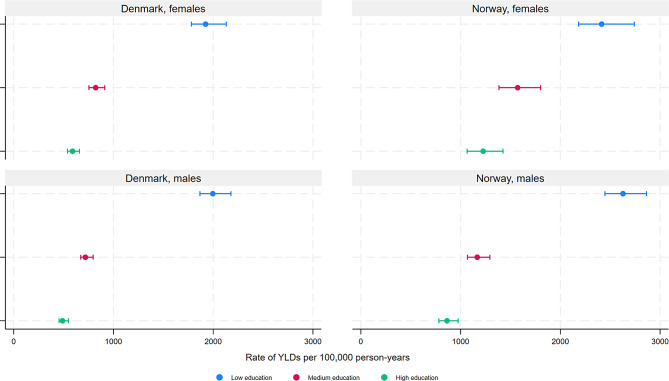
Years lived with disability due to any mental or substance use disorder by educational level, among patients registered in secondary health care in Denmark and Norway

**Fig. 2:**
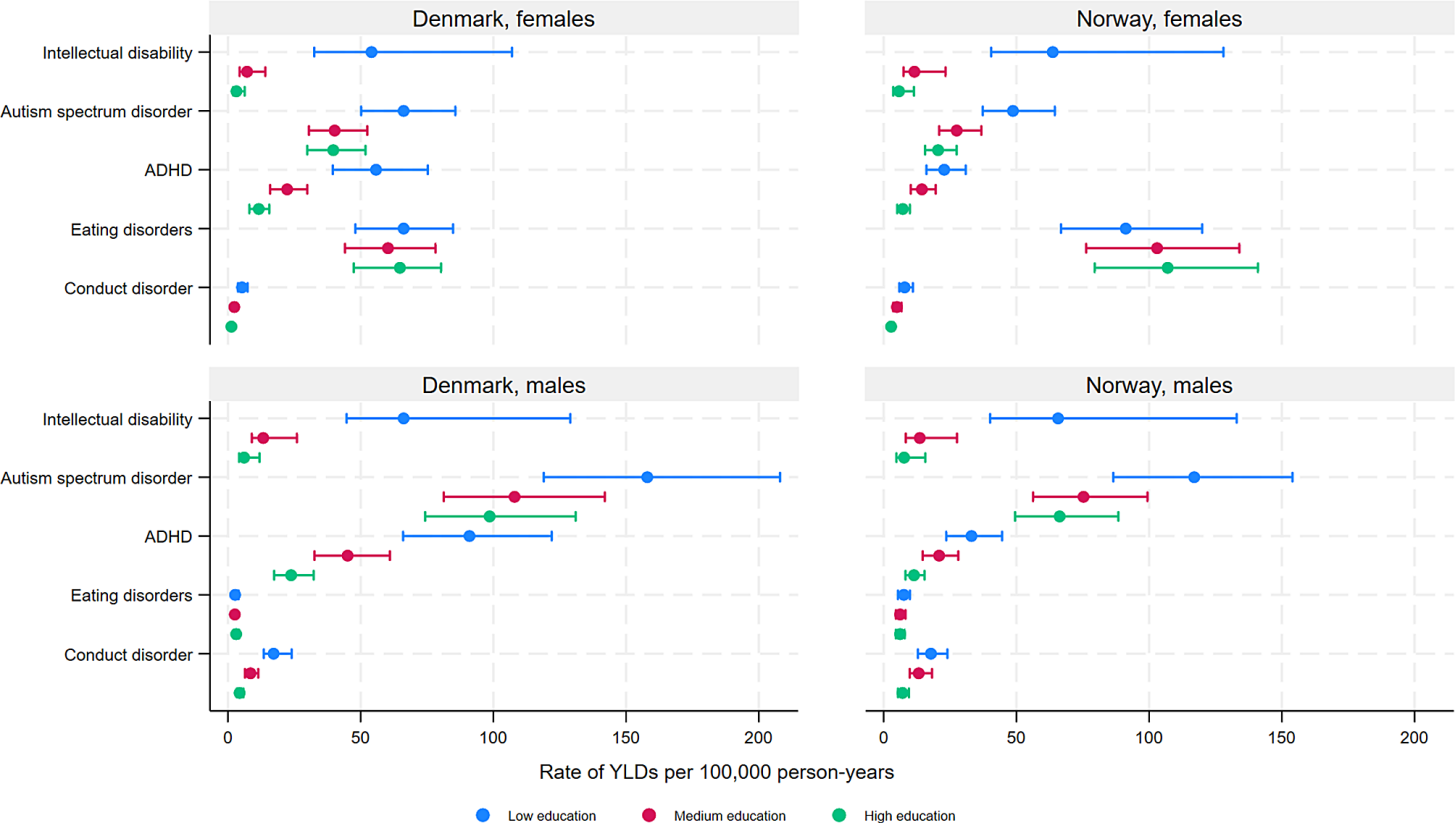
Years lived with disability due to mental disorders with onset in childhood by educational level, among patients registered in secondary health care in Denmark and Norway

**Fig. 3:**
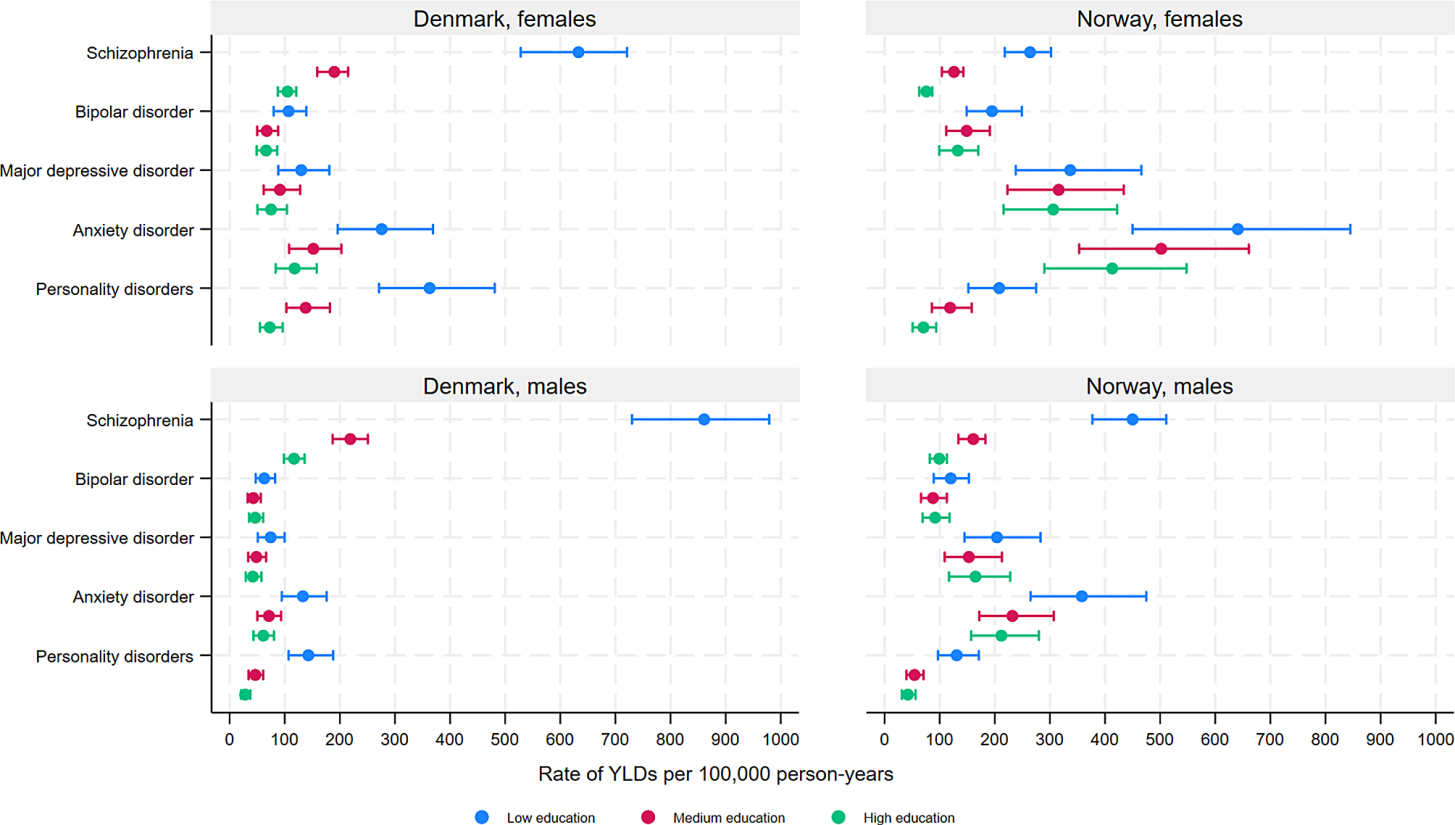
Years lived with disability due to mental disorders with onset in adulthood by educational level, among patients registered in secondary health care in Denmark and Norway

**Fig. 4:**
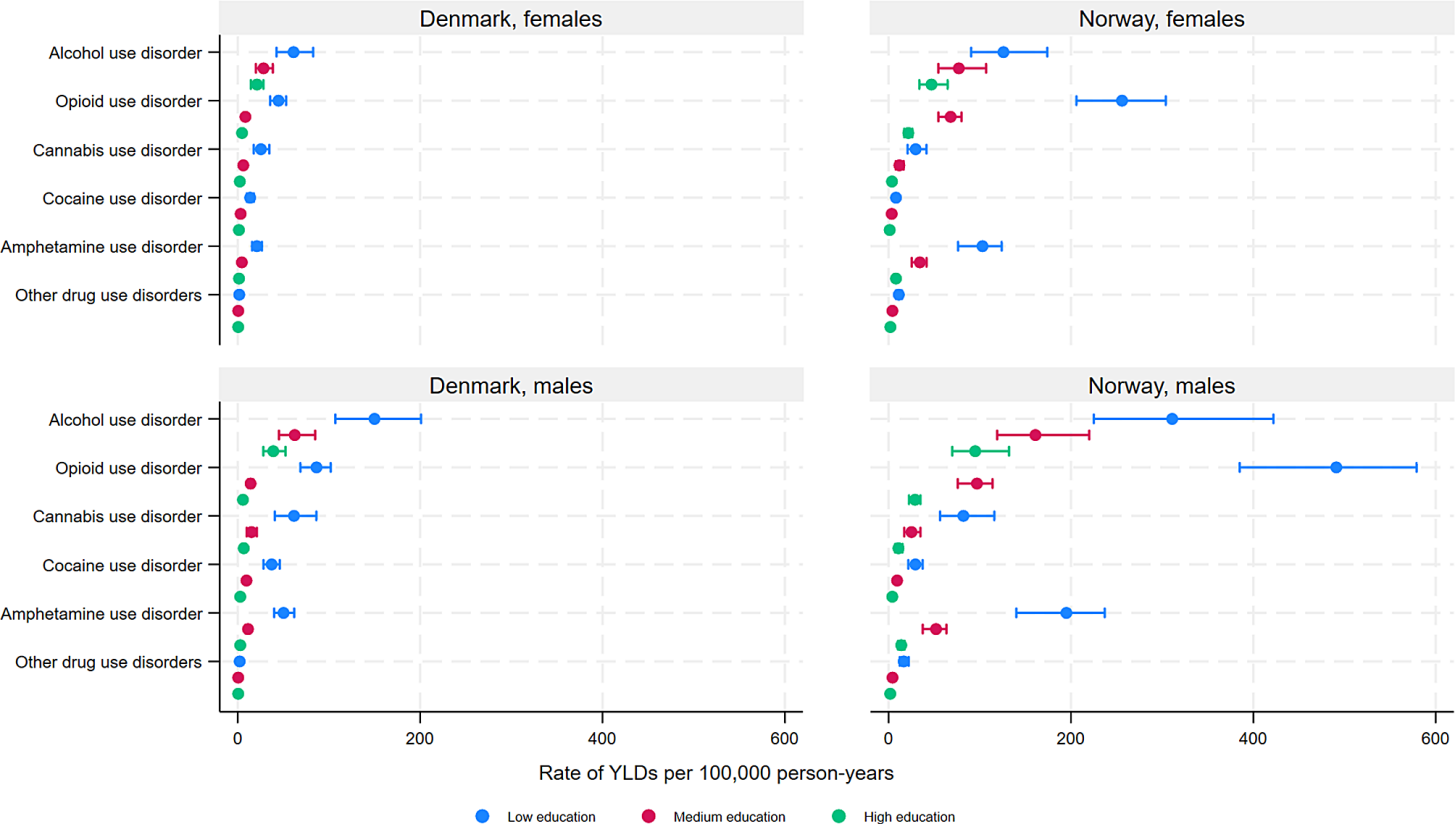
Years lived with disability (YLDs) due to substance use disorders by educational level, among patients registered in secondary health care in Denmark and Norway

In general, the overall age-standardised YLD rates for mental and substance use disorders were higher among individuals with low compared with medium and high educational level (Fig. 1, Supplementary Table 5) (Denmark males (ASR with 95% CIs for low, medium, high educational level): 1996 (1867–2178), 719 (672–796), 489 (455–548); Denmark females: 1924 (1781–2131), 822 (754–913), 590 (539–659); Norway males: 2628 (2447–2863), 1167 (1069–1293), 864 (782–975); Norway females: 2414 (2183–2741), 1572 (1385–1802), 1226 (1065–1425)), as well as for specific disorders, despite overlapping CIs between low and medium, and medium and high educational level (Figs. 1, 2, 3 and 4, Supplementary Table 5) for several disorders. However, overlapping CIs between low and high educational level were only found for ASD (except Norwegian females), eating disorders, bipolar disorder, MDD, and anxiety disorders (Norway only). The largest educational differences in YLD rates were found for intellectual disabilities (Fig. 2), schizophrenia (Fig. 3) and several substance use disorders (Fig. 4), among both males and females.

There were important similarities and notable differences between educational levels and sexes in terms of the top-three largest causes of YLDs (Figs. 2, 3 and 4). Schizophrenia was a top-three cause across all education -sex groups, except for females with medium or high education in Norway. For males with low education, AUD and opioid disorders in Norway, and ASD in Denmark, ranked among top-three. Among females, anxiety disorders and MDD were among the top-three causes across educational levels, as well as among males with high education. ASD was a top-three cause among males with high education in Denmark, and bipolar disorder was a top-three cause among females with high education in Norway (for all age-standardized rates, see supplementary Table 5).

**Fig. 5:**
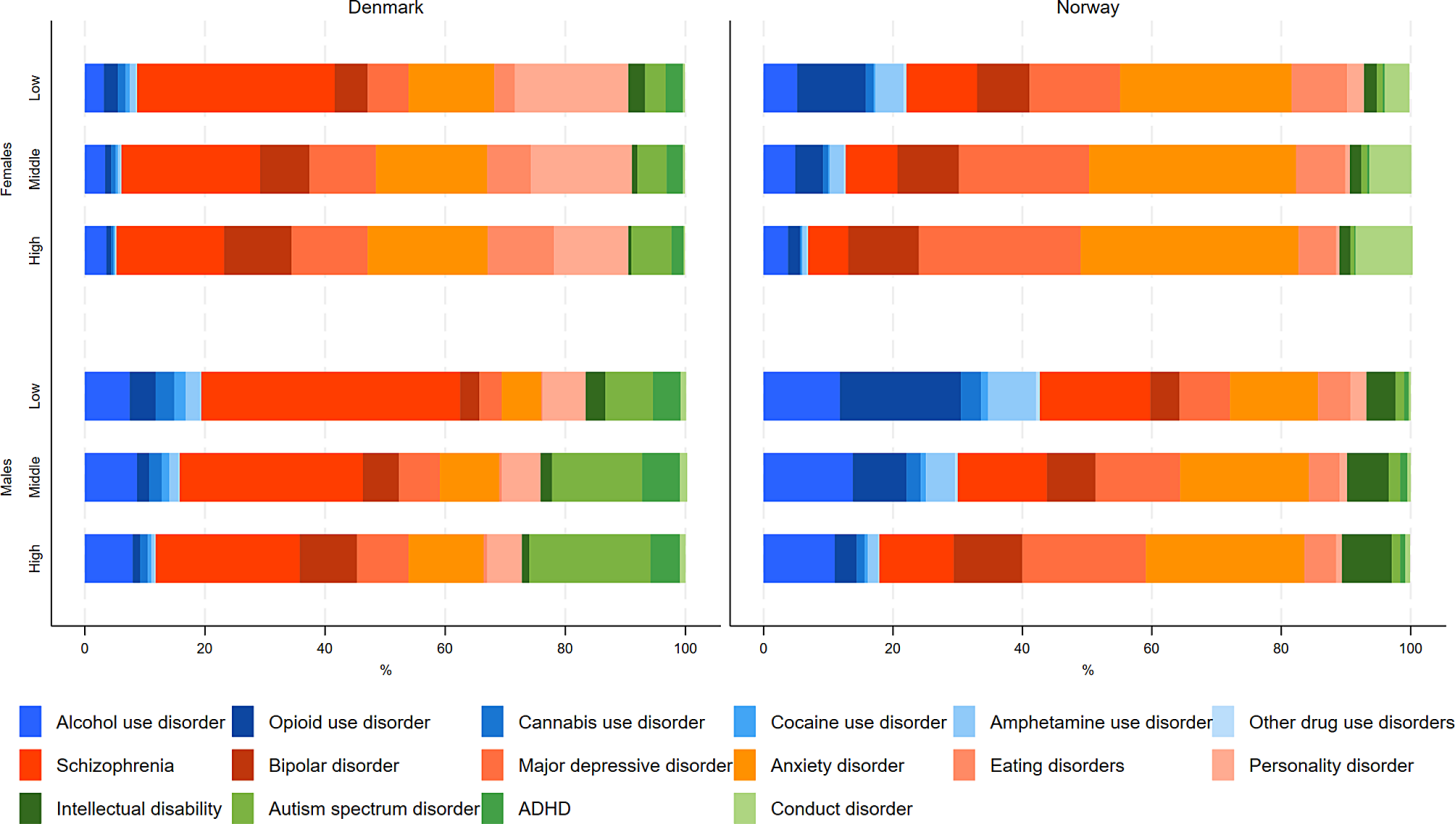
Proportion of total years lived with disability (YLDs) for mental disorders attributed to specific mental disorders by educational level among patients registered in secondary health care in Denmark and Norway

The contribution of each specific cause in terms of percentage of the total burden for mental and substance use disorders also differed by educational level and sex (Fig. 5, Supplementary Table 6). Although schizophrenia was a top-three contributor to YLDs across all strata, the attributed proportion decreased with higher educational levels, ranging from 6.2% (95% CI 4.8–7.4) of the total YLDs among females with high education, to 17.1% (95% CI 14.3–19.1) among males with low education in Norway, and from 17.9% (95% CI 14.6–20.6) among females with high education to 43.1% (95% CI 38.0-46.5) among males with low education in Denmark. In contrast, the contribution from the common mental disorders anxiety disorders and MDDs increased with educational level, ranging in Denmark from MDD causing 3.7% (95% CI 2.6–4.9) of the YLDs among low educated males, to anxiety disorders causing a fifth (20.0% (95% CI 14.7–25.2)) of the YLDs among females with high education. In Norway, the impact from common mental disorders ranged from 7.8% (95% CI 5.5–10.6) due to MDDs among low educated males, to anxiety disorders causing more than a third (33.7% (95% CI 25.4–40.6)) of the YLDs among high educated females. The contribution of each specific cause on total YLD rates in absolute numbers by educational level is presented in Supplementary Fig. 1.

## Discussion

In this registry-based cohort study, covering all residents of two welfare states, we found an inverse educational gradient in disease burden from most mental and substance disorders. The contribution of specific disorders to the total disease burden caused by mental and substance use disorders differed by educational level, with higher proportions attributed to substance use disorders, intellectual disability, and schizophrenia among those with lower educational levels and higher proportions attributed to anxiety disorders and MDD among those with higher educational levels.

While the GBD study has provided estimates on the disease burden from mental and substance use disorders by geography, age, and sex [1, 21, 22], and several studies have demonstrated an educational gradient in the prevalence of mental and substance use disorders [7, 9, 19, 39], this is, to the best of our knowledge, the first study to identify educational differences in non-fatal health loss (YLDs) due to mental and substance use disorders, providing new perspectives on the impact of mental disorders in the population. The results demonstrated a clear educational gradient, with between 2 and 4-fold higher overall YLD rates for mental and substance use disorders among those with low compared to high education, depending on sex. In contrast to studies focusing on describing educational differences in prevalence from mental and substance use disorders, our study also demonstrates how disorders with low prevalence, but high disability, can yield a substantial disease burden, particularly in populations with low educational levels. While schizophrenia ranked as the 7th and 14th most prevalent mental disorder diagnosis in the Danish and Norwegian secondary health care registries, respectively, it emerged as a leading disorder in terms of YLDs, across educational levels. There was further pronounced educational differences in absolute numbers of YLDs from this disorder, with the highest burden among those with low education. This was also the case for substance use disorders and intellectual disability, which showed a large educational gradient in absolute burden. In contrast, we found smaller educational differences in absolute YLD rates from MDD and anxiety disorders, in particular among females. The presence of other and more severe mental and substance use disorders among the groups with lower education, resulted in common mental disorders contributing less to the total non-fatal health loss in the low education groups compared to the groups with higher education.

Severe and chronic disorders with early onset were crucial contributors of disease burden among those with low educational levels. Although we cannot draw any causal conclusions based on this study, these findings may illustrate the bidirectional relationship between educational level and mental disorders [5, 6], in which presence of a mental and/or substance use disorder can affect the level of education an individual may achieve [15–18]. For instance, disorders such as schizophrenia often has its onset in young adulthood, a time when one is typically completing high school or initiating higher education. This may impact educational attainment and in part explain the observed educational gradient. Another example is intellectual disability, for which the largest educational gradient in disease burden was found. The diagnostic criteria for intellectual disabilities (codes F70-F79 in ICD-10) describes characteristics that may challenge the persons opportunities to achieve higher levels of education, in particular for the more severe states of these disorders. In addition, the chronic duration, and the shorter lifespan associated with intellectual disability, will further add to the burden from this condition.

On the other hand, education may provide access to important resources that can help prevent the development of mental and substance use disorders. Lower education is often related to poorer health literacy, less stable family- and partnerships, more disadvantaged living conditions, and fewer opportunities in the labour market (which in turn can increase the risk of long-term sick-leave, unemployment and poorer economy), all factors which can be associated with both health in general as well as mental health. Thus, the identified educational gradient in mental and substance use disorders may also be due to the additional strain experienced by persons with low compared to high education, which may lead to the development or worsening of mental health problems.

The higher burden of mental disorders found in groups with low educational levels, even in the egalitarian Nordic countries as represented here by Denmark and Norway, is concerning. In addition to the individual suffering, are mental and substance use disorders also associated with a high societal economic burden through productivity loss and health care spending [40–43]. Although studies also have shown inconsistent relationships for mental and substance use disorders across SEP-indicators (such as education, income, and race) [44, 45], low education may be an important indicator to be aware of. In any policy efforts to reduce socioeconomic inequalities in YLDs from these disorders, it is essential to view education as one of several correlated SEP-indicators. Thus, interventions should take into consideration the mechanisms involved in the relationship between education and disease burden from mental and substance use disorders, to be as targeted and effective as possible. This will require further research, preferably using large survey and registry-based linkages on individual and family level, to examine how socio-economic position and educational attainment affects the development of mental and substance use disorders and vice versa, and the associated disease burden, comorbidity with other health conditions, and premature death.

### Methodological considerations and limitations

The burden of disease concept is a summary measure of the health loss due to premature death and non-fatal health loss. In the ICD-10 system, mental disorders are rarely ascribed as an underlying cause of death. Hence, the disease burden from this group can almost exclusively be attributed to non-fatal health loss. In contrast, substance use disorders are important causes of premature death [25]. In the present analyses, we only included measures of non-fatal health loss. It is likely that if fatal health loss were included, the educational differences in disease burden from substance use disorders would be even greater.

Denmark and Norway provide excellent settings to examine the relationship between educational level and disease burden from mental and substance use disorders. Secondary and tertiary education, as well as access to secondary health care, are primarily universal and publicly funded in both countries. Thus, there should be less selection into education and health care use associated with for instance parental SEP in these countries than in many other high-income countries. A major strength of our study was the high-quality and complete linkage between population-based registry data on 7.2 million Danish and 6.2 million Norwegian residents, which eliminates the risk of loss to follow-up, self-reporting bias and duplicate records. Further, the registries provide information about causes rarely captured in health surveys, such as substance use disorders, bipolar disorder and schizophrenia. In addition, the use of individual-level data permitted precise adjustments for observed comorbidity of mental disorders and general medical conditions.

The present study also has important limitations. First, information on prevalence of mental and substance use disorders were based on recorded diagnoses from clinical settings, rather than from systematic well-described uniform assessments in the general population. Although many register-based diagnoses in Denmark and Norway have been confirmed to have good validity [46–51], the diagnoses cannot be taken as the true prevalence in the population, but rather reflect help-seeking behaviour and health care use. The potential underestimation of population prevalence of mental health problems by using diagnostic data from health records as proxy measures, may in particular be present in the part of the study period that included the COVID-19 pandemic, which is assumed to affect both the prevalence of mental health problems in the population [21], and the help seeking in the health services for these conditions [52]. Further, differences in prevalences between Denmark and Norway are likely due to differences in health service organisation, practice and the inclusion of different parts of the health services in the registries, and should not be interpreted as true differences between the countries (see supplementary material for more details). One important example where differences in prevalence must not be taken as true country differences, are substance use disorders, which are treated in different parts of the health system in Denmark and in Norway. In Denmark, the diagnoses in the present study were primarily taken from emergency rooms, or settings where they were recorded as comorbid to other mental or somatic disorders. In Norway, on the other hand, these disorders are treated in the secondary health care. Second, the registers do not provide information about recovery of the disorders, and therefore we applied remission rates from the GBD to model duration. These remission rates have not been validated within the GBD framework. However, because we used observed data, persons with recurrent episodes were less likely to be considered recovered of the disorder of interest compared with persons with fewer episodes. Third, we used average disability weights based on severity distributions; many of which were from the GBD studies. These distributions were not specific to Denmark and Norway, and can lead to both under- and overestimation of YLDs [53]. Fourth, because we included all age groups in the study, the increase in the average years of completed education observed since the 1970’s [54] will lead to higher educational level among the younger study population. Fifth, around 40% of the study population were below age 25 during the study period. To avoid linking all disease burden to low and middle education groups in children and adolescents, and in line with other studies [9, 55, 56], the highest educational level between children and parents were employed for this population. In particular for cases of mental disorders with primarily onset in childhood, this methodological choice may mean that the educational distribution of burden may be different in the population below than the population above age 25. One example of this is intellectual disability, where YLDs were also found in the group with high educational level, which in many cases are likely to be due to classification based on parental education level. Sixth, the choice of basing the categorisation of disorders on the categorisation used by GBD, means that Alzheimers disease and other dementias are not included in the present study, as these are categorised as neurological disorders in the GBD hierarchical systems. As a result, a large source of disease burden related to mental disorders, which in particular affects the older part of the population, is not accounted for in the present analyses. Finally, due to the cross-sectional design, and because the information about age of onset was based on when the diagnosis first appeared in the registry, the direction of causality cannot be determined in this study.

## Conclusion

The results suggest a pronounced inverse educational gradient in non-fatal disease burden from mental and substance use disorders, where most of disease burden related to these disorders falls on those with the fewest years of education. This should be taken into consideration when public health targets aimed at improving mental health and reducing social inequalities in health are developed and implemented.

## Electronic supplementary material

Below is the link to the electronic supplementary material.

Supplementary Material 1

## Data Availability

The data used in this study are stored on and accessed via the secure governmental servers of Statistics Denmark (https://www.dst.dk/) and University of Oslo (https://www.uio.no/english/services/it/research/sensitive-data/index.html). Danish and Norwegian data protection regulations and GDPR impose restrictions on sharing of individual data. Hence, there restrictions apply to the availability of these data, which were used under license for the current study, and so are not publicly available. The data that support the findings of this study are available from Statistics Denmark and/or the Danish Health Data Authority, and from Statistics Norway and the Norwegian Institute of Public Health. Data are however available from the authors upon reasonable request and with permission from the Regional Committee for Medical and Health Research Ethics and the data owners (Statistics Norway and the Norwegian Institute of Public Health), as well as from the Danish Data Protection Agency, Statistics Denmark and the Danish Health Data Authority.

## Abbreviations

ASR: Age standardised rates
ASD: Autism spectrum disorders
AUD: Alcohol use disorder
CI: Confidence intervals
DW: Disability weights
GBD: The Global Burden of Disease study
GMC: General medication conditions
ICD 10: International Classification of Diseases 10th version
ISCED: International Standard Classification of Education
MDD: Major depressive disorder
NPR: Norwegian National Patient Registry
PCRR: Danish Psychiatric Central Research Register
SEP: Socioeconomic position
SUD: Substance use disorders
YLD: Years lived with disability
