# Supplementary material for: Educational differences in years lived with disability due to mental and substance use disorders: a cohort study using nationwide Norwegian and Danish registries
Source: BMC Public Health. 2024 Sep 20;24:2576. doi: 10.1186/s12889-024-20064-0 (PMC11416009; doi:10.1186/s12889-024-20064-0)
Supplement: Supplementary file 1 — Supplementary Material 1 [file 12889_2024_20064_MOESM1_ESM.docx]

# Supplementary material to ”Educational differences in years lived with disability due to mental and substance use disorders: a cohort study using nationwide Norwegian and Danish registries”

## Study population

We included all residents, this includes immigrants with temporary or permanent residence, and citizens. In Denmark, they needed to have an active CPR number, which is a unique person number given to all people taking up residence in Denmark, whether it be temporary or permanent. In Norway, only persons who intend to live in the country for a longer period is given a person number, hence short-term residents are less likely to be included in the Norwegian sample. The Norwegian National Population Register has information on place of residence on January 1^st^ of each year, but no immigration or emigration dates. To determine precise follow-up time for each individual, we derived a random date for start of entry into the cohort in the year prior to first registration of place of residence and a random date for cohort exit in the final year of registration of place of residence. In addition, 4296 individuals (0.07%) were excluded due to data inconsistencies.

## Assessment of eating disorders:

In the GBD studies, estimates for anorexia and bulimia are presented separately. However, due to the structure of the Norwegian data, we could not identify specific eating disorders and grouped all ICD-10 F50 cases together. As the remission rates differed substantially between anorexia and bulimia, we calculated a weighted remission rate for eating disorders combined. The weights were the proportion of anorexia and bulimia cases by five-year age groups and sex in the Danish secondary care data. The disability weights (DW) for anorexia and bulimia were similar (DW_AN_ = 0.224, DW_BU_ = 0.223), hence we used the DW for anorexia as the DW for eating disorders.

## Methodological considerations

In general, the period prevalence for mental disorders diagnosed in secondary health care were higher in Norway compared with Denmark, except for autism spectrum disorder, personality disorders and schizophrenia, which were more commonly diagnosed in secondary health care in Denmark. We ascertained mental disorder cases in Denmark using the Danish Psychiatric Central Research Register which covers inpatient, outpatient or emergency room visits at psychiatric departments in hospitals (1). In contrast, the Norwegian National Patient Register has a larger coverage by also including contacts to specialised interdisciplinary addiction treatment and some specialists in addition to the same services as the Danish register (2). At the same time, we did not include primary health care data and in many high-income countries a large proportion of mental disorders among adults are treated only in primary health care (3, 4). Further, prevalence estimates can be affected by health spending prioritization. Norway has more direct costs related to mental health services compared to Denmark, 1.43 % of gross domestic product (GDP) vs 1.26% of GDP (38). Thus, it is likely that we have underestimated the true burden of mental and substance use disorders although the extent to which this will differ by educational level is not known.

**Supplementary table 1.** International Classification of Diseases, 10^th^ version (ICD-10) codes, minimum age of onset and disability weights for mental and substance use disorders.

| Disorder | ICD-10 codes | Earliest age of onset (Years) | Disability weight |
| --- | --- | --- | --- |
| Alcohol use disorder | F10 | 10 | 0.18 (0.11-0.27) |
| Opioid use disorder | F11 | 10 | 0.54 (0.39-0.67) |
| Cannabis use disorder | F12 | 10 | 0.07 (0.04-0.11) |
| Cocaine use disorder | F14 | 10 | 0.30 (0.20-0.40) |
| Amphetamine use disorder | F15 | 10 | 0.31 (0.21-0.41) |
| Other drug use disorders | F13, F16, F18 | 10 | 0.12 (0.07-0.17) |
| Schizophrenia | F20 | 10 | 0.71 (0.56-0.84) |
| Bipolar disorder | F30, F31 | 10 | 0.22 (0.15-0.31) |
| Major depressive disorder | F32, F33 | 10 | 0.25 (0.15-0.38) |
| Anxiety disorders | F40, F41, F42, F93 | 1 | 0.15 (0.09-0.21) |
| Personality disorder | F60 | 10 | 0.15 (0.10-0.21) |
| Eating disorders | F50 | 1 | 0.22 (0.15-0.31) |
| Idiopathic developmental  intellectual disability | F70-F79 | 1 | 0.07 (0.03-0.15) |
| Autism spectrum disorder | F84 | 1 | 0.17 (0.11-0.24) |
| ADHD | F90 | 1 | 0.05 (0.03-0.07) |
| Conduct disorder | F91 | 1 | 0.24 (0.16-0.34) |

**Supplementary table 2**. ICD-10 codes and associated disability weight^1^ for comorbid general medical conditions.

| **Category** | **Disorder** | **ICD-10 codes** | **Disability weight** |
| --- | --- | --- | --- |
| Circulatory system | Hypertension | I10-I13, I15 | 0.05 (0.03-0.07) |
|  | Dyslipidemia | E78 | 0.05 (0.03-0.07) |
|  | Ischemic heart disease | I20-I25 | 0.11 (0.07-0.15) |
|  | Atrial fibrillation | I48 | 0.22 (0.15-0.33) |
|  | Heart failure | I50 | 0.09 (0.06-0.13) |
|  | Peripheral artery occlusive disease | I70-I74 | 0.01 (0.01-0.03) |
|  | Ischemic stroke | I60-I64, I69 | 0.10 (0.05-0.18) |
| Endocrine | Diabetes | E10-E14 | 0.08 (0.05-0.11) |
|  | Thyroid disorder | E00-E07 | 0.07 (0.04-0.10) |
|  | Gout | E79, M10 | 0.02 (0.01-0.03) |
| Pulmonary system and allergy | Asthma | J45-J46 | 0.07 (0.04-0.10) |
|  | COPD | J40-J44, J47 | 0.13 (0.08-0.18) |
|  | Allergy | J30, L23, L50.0, T78.0. T78.2, T78.4 | 0.22 (0.14-0.31) |
| Gastrointestinal system | Ulcer/chronic gastritis | K221, K25-K28, K293-K295 | 0.07 (0.04-0.09) |
|  | Chronic liver disease | B16-B19, K70, K74, K766, I85 | 0.18 (0.12-0.24) |
|  | Inflammatory bowel disease | K50-K51 | 0.23 (0.16-0.32) |
|  | Diverticular disease of intestine | K57 | 0.05 (0.03-0.07) |
| Urogenital system | Chronic kidney disease | N03, N11, N18-N19 | 0.02 (0.01-0.02) |
|  | Prostate disorders | N40 | 0.07 (0.04-0.1,) |
| Musculoskeletal system | Connective tissue disorders | M05-M06, M08-M09, M30-M36, D86 | 0.13 (0.10-0.16) |
|  | Osteoporosis | M80-M82 | 0.13 (0.10-0.16) |
|  | Backpain | M54 | 0.16 (0.10-0.22) |
| Hematological system | HIV/AIDS | B20-B24 | 0.05 (0.03-0.07) |
|  | Anemias | D50-D53, D55-D59, D60-D61, D63-D64 | 0.03 (0.02-0.04) |
| Cancers | Cancer | C00-C43, C45-C97 | 0.11 (0.10-0.11) |
| Neurological system | Vision problems | H40, H25, H54 | 0.09 (0.06-0.12) |
|  | Hearing problems | H90-H91, H931 | 0.03 (0.02-0.05) |
|  | Migraine | G43 | 0.04 (0.02-0.07) |
|  | Epilepsy | G40-G41 | 0.27 (0.18-0.36) |
|  | Parkinson's disease | G20-G22 | 0.17 (0.11-0.23) |
|  | Multiple sclerosis | G35 | 0.37 (0.25-0.49) |
|  | Neuropathies | G50-G64 | 0.05 (0.03-0.07) |
|  | Dementia | F00-F03, G30-G31 | Weight depending on age and sex (Supplementary table 3) |

**Supplementary table 3.** Disability weights for dementia, by age and sex.

|  | Age | Disability weight (95% CI) |
| --- | --- | --- |
| **Females** | 35-39 | 0.20 (0.13-0.27) |
|  | 40-44 | 0.20 (0.13-0.27) |
|  | 45-49 | 0.21 (0.14-0.28) |
|  | 50-54 | 0.21 (0.14-0.29) |
|  | 55-59 | 0.22 (0.15-0.30) |
|  | 60-64 | 0.23 (0.15-0.31) |
|  | 65-69 | 0.24 (0.16-0.32) |
|  | 70-74 | 0.25 (0.17-0.34) |
|  | 75-79 | 0.24 (0.16-0.33) |
|  | 80-84 | 0.26 (0.18-0.35) |
|  | 85-89 | 0.28 (0.19-0.38) |
|  | 90-94 | 0.30 (0.20-0.40) |
|  | 95- | 0.32 (0.21-0.43) |
| **Males** | 35-39 | 0.18 (0.12-0.25) |
|  | 40-44 | 0.18 (0.12-0.25) |
|  | 45-49 | 0.19 (0.13-0.25) |
|  | 50-54 | 0.19 (0.13-0.26) |
|  | 55-59 | 0.20 (0.13-0.27) |
|  | 60-64 | 0.21 (0.14-0.28) |
|  | 65-69 | 0.22 (0.15-0.29) |
|  | 70-74 | 0.22 (0.15-0.30) |
|  | 75-79 | 0.22 (0.15-0.30) |
|  | 80-84 | 0.24 (0.16-0.32) |
|  | 85-89 | 0.26 (0.17-0.35) |
|  | 90-94 | 0.28 (0.19-0.37) |
|  | 95- | 0.30 (0.20-0.40) |

Supplementary table 4. Cases of mental disorders (numbers and percent (%) of total population diagnosed) in 2011-2021 in secondary health care in Denmark and Norway, stratified by educational level (low, medium, high).

|  | **Denmark** | | | **Norway** | | |
| --- | --- | --- | --- | --- | --- | --- |
|  | **Low** | **Medium** | **High** | **Low** | **Medium** | **High** |
| **Any mental or substance use disorder** | 110,671 (8.49) | 187,817 (6.33) | 108,567 (4.78) | 159,537 (14.55) | 272,551 (11.84) | 239,926 (9.83) |
| **Mental disorders with onset in childhood** |  |  |  |  |  |  |
| Intellectual disability | 5921 (0.45) | 9940 (0.34) | 5738 (0.25) | 11,173 (1.02) | 7297 (0.32) | 5939 (0.24) |
| Autism spectrum disorder | 14,115 (1.08) | 23,687 (0.80) | 13,503 (0.60) | 5008 (0.46) | 10,495 (0.46) | 12,660 (0.52) |
| ADHD | 20,905 (1.60) | 35,347 (1.19) | 20,328 (0.90) | 23,147 (2.11) | 45,352 (1.97) | 33,452 (1.37) |
| Eating disorders | 6030 (0.46) | 10,344 (0.35) | 6017 (0.27) | 3378 (0.31) | 10,328 (0.45) | 13,953 (0.57) |
| Conduct disorder | 1099 (0.08) | 2000 (0.07) | 1211 (0.05) | 1257 (0.11) | 3006 (0.13) | 2323 (0.10) |
| **Mental disorders with onset in adulthood** |  |  |  |  |  |  |
| Schizophrenia | 9695 (0.74) | 16,392 (0.55) | 9509 (0.42) | 7487 (0.68) | 7283 (0.32) | 4518 (0.19) |
| Bipolar disorder | 7677 (0.59) | 13,067 (0.44) | 7538 (0.33) | 10,095 (0.92) | 18,271 (0.79) | 16,469 (0.67) |
| Major depressive disorder | 38,504 (2.95) | 65,619 (2.21) | 37,806 (1.67) | 59,981 (5.47) | 115,345 (5.01) | 109,994 (4.51) |
| Anxiety disorder | 28,241 (2.17) | 48,123 (1.62) | 27,713 (1.22) | 55,031 (5.02) | 101,747 (4.42) | 95,556 (3.91) |
| Personality disorder | 16,533 (1.27) | 27,899 (0.94) | 16,195 (0.71) | 15,513 (1.41) | 22,258 (0.97) | 16,146 (0.66) |
| **Substance use disorders** |  |  |  |  |  |  |
| Alcohol use disorder | 13,195 (1.01) | 22,056 (0.74) | 12,607 (0.56) | 30,704 (2.80) | 44,041 (1.91) | 23,449 (0.96) |
| Opioid use disorder | 1379 (0.11) | 2323 (0.08) | 1345 (0.06) | 11,606 (1.06) | 7513 (0.33) | 2604 (0.11) |
| Cannabis use disorder | 7039 (0.54) | 11,700 (0.39) | 6896 (0.30) | 11,609 (1.06) | 12,911 (0.56) | 7390 (0.30) |
| Cocaine use disorder | 1271 (0.10) | 2077 (0.07) | 1220 (0.05) | 1207 (0.11) | 1512 (0.07) | 840 (0.03) |
| Amphetamine use disorder | 1456 (0.11) | 2352 (0.08) | 1443 (0.06) | 9081 (0.83) | 7747 (0.34) | 2907 (0.12) |
| Other drug use disorders | 1649 (0.13) | 2564 (0.09) | 1523 (0.07) | 11,288 (1.03) | 9952 (0.43) | 4537 (0.19) |

**Supplementary table 5.** Age-standardized rates (ASR) of years lived with disability (YLDs) and corresponding 95% confidence interval (95% CI) for mental disorders and substance use disorders in Danish and Norwegian secondary health care, females and males, stratified by educational level.

|  | **Educational level** | **Females** | | **Males** | |
| --- | --- | --- | --- | --- | --- |
| **Disorder** |  | **Denmark** | **Norway** | **Denmark** | **Norway** |
|  |  | **ASR (95% CI)** | **ASR (95% CI)** | **ASR (95% CI)** | **ASR (95% CI)** |
| Any mental disorder | Low | 1924 (1781-2131) | 2414 (2183-2741) | 1996 (1867-2178) | 2628 (2447-2863) |
|  | Medium | 822 (754-913) | 1572 (1385-1802) | 719 (672-796) | 1167 (1069-1293) |
|  | High | 590 (539-659) | 1226 (1065-1425) | 489 (455-548) | 864 (782-975) |
| Mental disorders with onset in childhood | | | | | |
| Eating disorders | Low | 66.2 (48.0-84.8) | 91.2 (66.8-120) | 2.7 (2.1-4) | 7.6 (5.4-9.9) |
|  | Medium | 60.3 (44.1-78.2) | 103 (76.3-134) | 2.6 (2.0-3.5) | 6.2 (4.6-8.2) |
|  | High | 64.8 (47.4-80.3) | 107 (79.5-141) | 3.1 (2.2-4.2) | 6.2 (4.6-7.9) |
| Intellectual disability | Low | 54.1 (32.5-107) | 63.7 (40.5-128) | 66.2 (44.7-129) | 65.7 (40.1-133) |
|  | Medium | 7.2 (4.4-14.1) | 11.6 (7.5-23.3) | 13.3 (9.0-26.0) | 13.6 (8.3-27.6) |
|  | High | 3.2 (2.0-6.3) | 5.8 (3.6-11.4) | 6.1 (4.2-11.9) | 7.7 (4.8-15.7) |
| Autism spectrum disorder | Low | 66.2 (50.2-85.7) | 48.7 (37.3-64.5) | 158 (119-208) | 117 (86.5-154) |
|  | Medium | 40.2 (30.5-52.5) | 27.5 (20.9-36.8) | 108 (81.3-142) | 75.3 (56.3-99.4) |
|  | High | 39.7 (29.9-51.8) | 20.5 (15.6-27.5) | 98.6 (74.3-131) | 66.3 (49.5-88.4) |
| ADHD | Low | 55.8 (39.5-75.3) | 22.8 (16.1-30.9) | 91.0 (66.0-122) | 33.1 (23.6-44.6) |
|  | Medium | 22.3 (15.9-29.9) | 14.4 (10.2-19.6) | 45.1 (32.6-61.0) | 20.9 (14.7-28.1) |
|  | High | 11.6 (8.1-15.6) | 7.2 (5.1-9.9) | 23.8 (17.4-32.3) | 11.4 (8.2-15.4) |
| Conduct disorder | Low | 5.3 (3.8-7.4) | 7.9 (5.9-11) | 17.2 (13.5-24.0) | 17.8 (12.9-24) |
|  | Medium | 2.4 (1.7-3.2) | 5.0 (3.6-6.7) | 8.5 (6.4-11.4) | 13.2 (9.8-18.2) |
|  | High | 1.3 (1.0-1.8) | 2.8 (2.1-3.8) | 4.4 (3.3-5.8) | 7.1 (5.4-9.5) |
| Mental disorders with onset in adulthood | | | | | |
| Schizophrenia | Low | 633 (528-721) | 264 (218-302) | 861 (730-979) | 450 (377-511) |
|  | Medium | 190 (159-215) | 126 (104-143) | 219 (187-251) | 161 (134-183) |
|  | High | 105 (87.6-121) | 75.8 (62.9-86.5) | 117 (98.7-136) | 99.3 (82.0-113) |
| Bipolar disorder | Low | 107 (80.0-139) | 195 (149-249) | 62.9 (47.3-82.5) | 120 (89.2-153) |
|  | Medium | 67.4 (50.0-88.0) | 149 (112-191) | 43.1 (32.7-56.6) | 88.0 (66.1-113) |
|  | High | 66.2 (49.1-86.3) | 133 (99.2-170) | 46.6 (35.3-61.0) | 91.8 (69.0-118) |
| Major depressive disorder | Low | 130 (88.4-181) | 337 (238-466) | 74.4 (51.1-99.7) | 204 (145-283) |
|  | Medium | 91.4 (61.7-128) | 316 (223-434) | 48.5 (33.8-66.2) | 153 (109-213) |
|  | High | 75.2 (50.6-104) | 306 (216-422) | 42.2 (29.3-57.7) | 165 (117-228) |
| Anxiety disorder | Low | 276 (196-369) | 641 (450-845) | 133 (94.8-176) | 358 (265-475) |
|  | Medium | 152 (108-203) | 502 (353-661) | 71.3 (50.4-93.4) | 232 (172-307) |
|  | High | 118 (83.6-158) | 413 (290-548) | 61.2 (43.3-80.5) | 212 (157-280) |
| Personality disorder | Low | 363 (271-481) | 208 (152-275) | 143 (107-188) | 131 (96.9-171) |
|  | Medium | 138 (103-182) | 119 (85.9-158) | 46.6 (34.5-61.0) | 54.4 (39.6-70.7) |
|  | High | 72.9 (55.0-96.3) | 70.6 (50.9-93.7) | 28.4 (20.8-37.6) | 42.3 (31.3-56.1) |
| Substance use disorders | | | | | |
| Alcohol use disorder | Low | 61.2 (42.5-82.7) | 126 (90.5-174) | 150 (107-201) | 311 (225-422) |
|  | Medium | 28.3 (19.9-38.5) | 77.0 (54.5-107) | 62.5 (45.2-84.9) | 161 (119-220) |
|  | High | 21.1 (14.4-28.1) | 47.0 (33.8-64.8) | 39.0 (28.0-52.4) | 95 (69.8-132) |
| Opioid use disorder | Low | 44.7 (35.6-53) | 256 (206-304) | 86.4 (68.7-102) | 491 (385-579) |
|  | Medium | 8.4 (6.5-10.0) | 68.0 (54.6-79.9) | 14.1 (11.3-16.7) | 96.9 (75.8-114) |
|  | High | 4.7 (3.5-5.9) | 21.6 (17.1-26.0) | 5.7 (4.1-7.1) | 29.0 (22.4-34.9) |
| Cannabis use disorder | Low | 25.4 (17.5-34.6) | 29.7 (20.8-41.5) | 61.7 (40.6-86.2) | 82.0 (56.4-116) |
|  | Medium | 6.1 (4.3-8.4) | 11.8 (7.9-16.4) | 15.1 (9.8-20.9) | 25.1 (17.1-34.9) |
|  | High | 2.3 (1.6-3.3) | 3.7 (2.5-5.1) | 6.6 (4.4-9.1) | 10.8 (7.2-15.2) |
| Cocaine use disorder | Low | 13.6 (10.0-17.6) | 8.1 (6.2-10.7) | 37.2 (28.2-46) | 29.4 (21.6-37.3) |
|  | Medium | 3.2 (2.3-4.0) | 3.4 (2.5-4.4) | 9.6 (7.3-11.8) | 9.3 (7.0-11.9) |
|  | High | 1.3 (0.9-1.6) | 1.2 (0.8-1.5) | 2.8 (2.1-3.6) | 4.1 (3.0-5.3) |
| Amphetamine use disorder | Low | 21 (15.7-26.4) | 103 (76.2-124) | 50.2 (39.9-61.9) | 195 (140-237) |
|  | Medium | 4.5 (3.2-5.4) | 34.3 (25.3-41.7) | 11.3 (8.4-13.6) | 52.0 (37.4-63.4) |
|  | High | 1.4 (1.0-1.8) | 8.1 (6.0-10.0) | 2.8 (2.0-3.5) | 13.9 (10.2-17.6) |
| Other drug use disorders | Low | 1.7 (1.1-2.3) | 11.2 (8.1-14.6) | 2.1 (1.6-2.8) | 16.6 (12.2-21.9) |
|  | Medium | 0.6 (0.4-0.8) | 4.2 (3.1-5.5) | 0.5 (0.4-0.7) | 4.3 (3.2-5.7) |
|  | High | 0.5 (0.3-0.7) | 2.0 (1.4-2.6) | 0.5 (0.3-0.6) | 1.8 (1.3-2.4) |

**Supplementary table 6**. Proportion (percentage (%) with 95% confidence intervals (95% CI) of total years lived with disability (YLDs) for mental and substance use disorders attributed to specific disorders by educational level among females and males in secondary health care Denmark and Norway

|  |  | **Females** | | | **Males** | | |
| --- | --- | --- | --- | --- | --- | --- | --- |
| **Disorder** | **Country** | **Low** | **Middle** | **High** | **Low** | **Middle** | **High** |
|  |  | **% (95% CI)** | **% (95% CI)** | **% (95% CI)** | **% (95% CI)** | **% (95% CI)** | **% (95% CI)** |
| Mental disorders with onset in childhood | | | | | | | |
| Intellectual disability | Denmark | 2.8 (1.7-5.5) | 0.9 (0.5-1.7) | 0.5 (0.3-1.1) | 3.3 (2.2-6.2) | 1.9 (1.3-3.4) | 1.2 (0.8-2.3) |
|  | Norway | 2.0 (1.5-2.7) | 1.8 (1.3-2.3) | 1.7 (1.2-2.3) | 4.5 (3.4-5.8) | 6.4 (4.9-8.3) | 7.7 (5.7-10) |
| Autism spectrum disorder | Denmark | 3.4 (2.6-4.5) | 4.9 (3.6-6.3) | 6.7 (5.1-8.7) | 7.9 (5.9-10.2) | 15.0 (11.4-19.1) | 20.2 (15.5-25.2) |
|  | Norway | 0.9 (0.7-1.3) | 0.9 (0.6-1.3) | 0.6 (0.4-0.8) | 1.3 (0.9-1.7) | 1.8 (1.3-2.4) | 1.3 (0.9-1.8) |
| ADHD | Denmark | 2.9 (2.0-3.9) | 2.7 (1.9-3.6) | 2.0 (1.4-2.6) | 4.6 (3.3-6.1) | 6.3 (4.6-8.3) | 4.9 (3.6-6.5) |
|  | Norway | 0.3 (0.2-0.5) | 0.3 (0.2-0.4) | 0.2 (0.2-0.3) | 0.7 (0.5-0.9) | 1.1 (0.8-1.5) | 0.8 (0.6-1.1) |
| Conduct disorder | Denmark | 0.3 (0.2-0.4) | 0.3 (0.2-0.4) | 0.2 (0.2-0.3) | 0.9 (0.7-1.2) | 1.2 (0.9-1.6) | 0.9 (0.7-1.2) |
|  | Norway | 3.8 (2.8-5.0) | 6.5 (4.8-8.6) | 8.8 (6.4-11.5) | 0.3 (0.2-0.4) | 0.5 (0.4-0.7) | 0.7 (0.5-0.9) |
| Mental disorders with onset in adulthood | | | | | | | |
| Schizophrenia | Denmark | 32.9 (27.7-36.6) | 23.1 (19.3-26.2) | 17.9 (14.6-20.6) | 43.1 (38.0-46.5) | 30.5 (26.2-33.7) | 24 (20.2-27.3) |
|  | Norway | 10.9 (8.7-12.7) | 8.0 (6.3-9.6) | 6.2 (4.8-7.4) | 17.1 (14.3-19.1) | 13.8 (11.2-15.7) | 11.5 (9.0-13.2) |
| Bipolar disorder | Denmark | 5.5 (4.1-7.2) | 8.2 (6.0-10.5) | 11.2 (8.3-14.4) | 3.2 (2.4-4.2) | 6.0 (4.5-7.7) | 9.5 (7.1-12.1) |
|  | Norway | 8.1 (6.0-10.2) | 9.5 (7.0-12.1) | 10.9 (8.0-13.8) | 4.5 (3.3-5.9) | 7.5 (5.6-9.6) | 10.6 (7.8-13.5) |
| Major depressive disorder | Denmark | 6.8 (4.7-9.2) | 11.1 (7.8-14.9) | 12.7 (8.9-16.8) | 3.7 (2.6-4.9) | 6.8 (4.7-9.0) | 8.6 (6.0-11.4) |
|  | Norway | 14.0 (10.0-18.5) | 20.1 (14.8-26.3) | 25.0 (18.5-32.5) | 7.8 (5.5-10.6) | 13.1 (9.4-17.4) | 19.1 (13.7-25.1) |
| Anxiety disorder | Denmark | 14.3 (10.5-18.2) | 18.5 (13.6-23.4) | 20.0 (14.7-25.2) | 6.7 (4.8-8.8) | 9.9 (7.0-12.8) | 12.5 (8.9-16.0) |
|  | Norway | 26.5 (19.7-32.2) | 32.0 (24.0-38.5) | 33.7 (25.4-40.6) | 13.6 (10.1-17.6) | 19.9 (15.1-25) | 24.5 (18.8-30.2) |
| Eating disorders | Denmark | 3.4 (2.4-4.4) | 7.3 (5.3-9.3) | 11.0 (8.0-13.8) | 0.1 (0.1-0.2) | 0.4 (0.3-0.5) | 0.6 (0.4-0.9) |
|  | Norway | 8.6 (6.3-11.5) | 7.6 (5.4-10.0) | 5.8 (4.1-7.7) | 5.0 (3.7-6.4) | 4.7 (3.4-5.9) | 4.9 (3.5-6.4) |
| Personality disorder | Denmark | 18.9 (14.3-23.8) | 16.8 (12.7-21.3) | 12.4 (9.2-16.2) | 7.2 (5.2-9.3) | 6.5 (4.7-8.5) | 5.8 (4.2-7.7) |
|  | Norway | 2.6 (1.6-5.2) | 0.7 (0.5-1.5) | 0.5 (0.3-0.9) | 2.5 (1.6-5.0) | 1.2 (0.7-2.4) | 0.9 (0.5-1.8) |
| Substance use disorders | | | | | | | |
| Alcohol use disorder | Denmark | 3.2 (2.2-4.3) | 3.4 (2.4-4.7) | 3.6 (2.4-4.8) | 7.5 (5.4-10) | 8.7 (6.3-11.4) | 8.0 (5.7-10.6) |
|  | Norway | 5.2 (3.7-7.2) | 4.9 (3.5-6.9) | 3.8 (2.7-5.5) | 11.8 (8.8-15.4) | 13.8 (10.2-17.9) | 11.0 (8.1-14.5) |
| Opioid use disorder | Denmark | 2.3 (1.8-2.8) | 1.0 (0.8-1.2) | 0.8 (0.6-1.0) | 4.3 (3.3-5.1) | 2.0 (1.5-2.3) | 1.2 (0.8-1.4) |
|  | Norway | 10.6 (8.4-12.7) | 4.3 (3.4-5.3) | 1.8 (1.3-2.2) | 18.7 (14.7-21.7) | 8.3 (6.4-9.8) | 3.4 (2.5-4.1) |
| Cannabis use disorder | Denmark | 1.3 (0.9-1.8) | 0.7 (0.5-1.0) | 0.4 (0.3-0.6) | 3.1 (2.1-4.3) | 2.1 (1.4-2.9) | 1.3 (0.9-1.9) |
|  | Norway | 1.2 (0.8-1.7) | 0.8 (0.5-1.1) | 0.3 (0.2-0.4) | 3.1 (2.1-4.3) | 2.2 (1.5-3.0) | 1.2 (0.8-1.7) |
| Cocaine use disorder | Denmark | 0.7 (0.5-0.9) | 0.4 (0.3-0.5) | 0.2 (0.1-0.3) | 1.9 (1.4-2.3) | 1.3 (1.0-1.7) | 0.6 (0.4-0.7) |
|  | Norway | 0.3 (0.2-0.4) | 0.2 (0.2-0.3) | 0.1 (0.1-0.1) | 1.1 (0.8-1.4) | 0.8 (0.6-1.0) | 0.5 (0.3-0.6) |
| Amphetamine use disorder | Denmark | 1.1 (0.8-1.4) | 0.5 (0.4-0.7) | 0.2 (0.2-0.3) | 2.5 (1.9-3.1) | 1.6 (1.1-1.9) | 0.6 (0.4-0.7) |
|  | Norway | 4.3 (3.1-5.1) | 2.2 (1.6-2.7) | 0.7 (0.5-0.8) | 7.4 (5.3-9.2) | 4.5 (3.2-5.6) | 1.6 (1.1-2.1) |
| Other drug use disorders | Denmark | 0.1 (0.1-0.1) | 0.1 (0.0-0.1) | 0.1 (0.1-0.1) | 0.1 (0.1-0.1) | 0.1 (0.1-0.1) | 0.1 (0.1-0.1) |
|  | Norway | 0.5 (0.3-0.6) | 0.3 (0.2-0.4) | 0.2 (0.1-0.2) | 0.6 (0.5-0.8) | 0.4 (0.3-0.5) | 0.2 (0.1-0.3) |

**
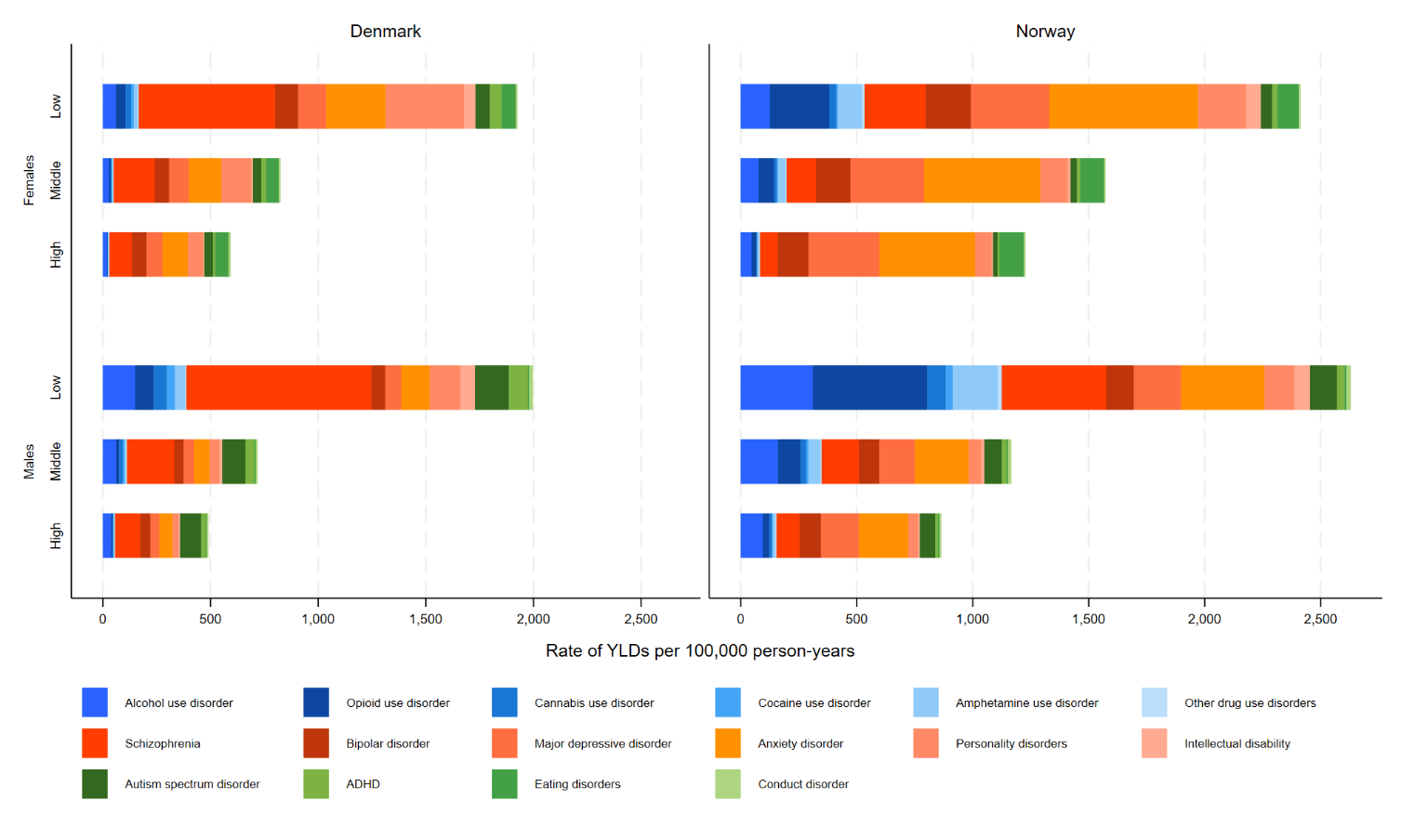
Supplementary Figure 1.** Age-standardised YLD rates for mental disorders and substance use disorders by educational level among patients registered in secondary health care in Denmark and Norway.

**References**

1. Mors O, Perto GP, Mortensen PB. The Danish Psychiatric Central Research Register. Scand J Public Health. 2011;39(7 Suppl):54-7.

2. Norwegian Directorate of Health. Norwegian Patient Registry - A Central Health Registry [Available from: <https://www.helsedirektoratet.no/tema/statistikk-registre-og-rapporter/helsedata-og-helseregistre/norsk-pasientregister-npr>.

3. Sundquist J, Ohlsson H, Sundquist K, Kendler KS. Common adult psychiatric disorders in Swedish primary care where most mental health patients are treated. Bmc Psychiatry. 2017;17(1):235.

4. Barbato A VM, Rapisarda F, Lora A, de Almeida JM,. EU compass for action on mental health and well-being. Access to mental health care in Europe. Scientific paper. Funded by the European Union in the frame of the 3rd EU Health Programme (2014–2020). 2016.
